# Sasang constitutional types for the risk prediction of metabolic syndrome: a 14-year longitudinal prospective cohort study

**DOI:** 10.1186/s12906-017-1936-4

**Published:** 2017-09-02

**Authors:** Sunghee Lee, Seung Ku Lee, Jong Yeol Kim, Namhan Cho, Chol Shin

**Affiliations:** 1 Department of Food and Nutrition, College of Health Science, Kangwon National University, Chuncheon, Gangwon Republic of Korea; 2 Institute of Human Genomic Study, College of Medicine, Korea University Ansan Hospital, Ansan, Republic of Korea; 3 Medical Research Division, Korea Institute of Oriental Medicine, Yuseong-gu, Republic of Korea; 4 Department of Preventive Medicine, Ajou University School of Medicine, Suwon, Republic of Korea; 5 Department of Pulmonary, Sleep and Critical Care Medicine, Department of Internal Medicine, Korea University Ansan Hospital, Ansan, Republic of Korea; 6 Institute of Human Genomic Study, Department of Internal Medicine, Ansan Hospital Korea University, 516 Gojan-1-dong, Danwon-gu, Ansan-si, Gyeonggi-do 425-707 South Korea

**Keywords:** Sasang constitutional types, Metabolic syndrome, Prediction

## Abstract

**Background:**

To examine whether the use of Sasang constitutional (SC) types, such as Tae-yang (TY), Tae-eum (TE), So-yang (SY), and So-eum (SE) types, increases the accuracy of risk prediction for metabolic syndrome.

**Methods:**

From 2001 to 2014, 3529 individuals aged 40 to 69 years participated in a longitudinal prospective cohort. The Cox proportional hazard model was utilized to predict the risk of developing metabolic syndrome.

**Results:**

During the 14 year follow-up, 1591 incident events of metabolic syndrome were observed. Individuals with TE type had higher body mass indexes and waist circumferences than individuals with SY and SE types. The risk of developing metabolic syndrome was the highest among individuals with the TE type, followed by the SY type and the SE type. When the prediction risk models for incident metabolic syndrome were compared, the area under the curve for the model using SC types was significantly increased to 0.8173. Significant predictors for incident metabolic syndrome were different according to the SC types. For individuals with the TE type, the significant predictors were age, sex, body mass index (BMI), education, smoking, drinking, fasting glucose level, high-density lipoprotein (HDL) cholesterol level, systolic and diastolic blood pressure, and triglyceride level. For Individuals with the SE type, the predictors were sex, smoking, fasting glucose, HDL cholesterol level, systolic and diastolic blood pressure, and triglyceride level, while the predictors in individuals with the SY type were age, sex, BMI, smoking, drinking, total cholesterol level, fasting glucose level, HDL cholesterol level, systolic and diastolic blood pressure, and triglyceride level.

**Conclusions:**

In this prospective cohort study among 3529 individuals, we observed that utilizing the SC types significantly increased the accuracy of the risk prediction for the development of metabolic syndrome.

## Background

Sasang constitutional medicine (SCM) is a Korean traditional medicine system that originated in 1894. SCM classifies human beings into four defined types, called Sasang constitutional (SC) types. Based on individual phenotypic characteristics such as body shape, personality, voice, and visceral organ functions [1], the four types, Tae-yang (TY), Tae-eum (TE), So-yang (SY), and So-eum (SE), were classified with different disease susceptibilities and treatment responses [1, 2]. Considering these specific susceptibilities of the SC types, different treatments for patients with the same disease are implemented. Previous studies related to the risk prediction model with the SC types have reported that the SC type was a strong indicator for type 2 diabetes [3] and cardiovascular diseases [4]. Recent studies have provided scientific evidence to prove vulnerable organs and related diseases for each SC type. According to the theory of classifying SC types, vulnerable hypoactive organs and the corresponding susceptible diseases were specified [1, 2, 5], confirmed with a recent systematic review with 15 studies [6]. For example, individuals with the TE type are considered to be vulnerable to lung-related diseases [1, 2, 5]. Additionally, individuals with the SY type have been known to have kidney as a vulnerable organ and have a greater risk of susceptible diseases such as urinary or renal diseases [1, 2]. Further, individuals with the SE type have been considered to have pancreas as a hypoactive organ and have an increased risk of gastrointestinal diseases [5].

Metabolic syndrome is diagnosed when at least three of the five following components are present: abdominal obesity, high blood pressure, decreased high density lipoprotein (HDL) cholesterol level, elevated triglyceride level, and impaired glucose tolerance [7]. Metabolic syndrome has been reported to increase the risk of developing type 2 diabetes, cardiovascular diseases, and all-cause mortality [8]. According to the National Health and Nutrition Examination Survey (NHANES) 2003–2012, the prevalence of metabolic syndrome was 35% for all adults and 50% for adults over 60 years of age [9]. Further assessing the prevalence by the SC type, individuals with the TE (Odds Ratio [OR] =4.52, 95% Confidence Intervals [CIs] 3.36, 6.07) and the SY (OR =2.00, 95% CI 1.47, 2.74) types illustrated a higher risk for metabolic syndrome than those with the SE type. The prevalence rates of metabolic syndrome for the TE, SY, and SE types were 48.85%, 30.59%, and 18.02%, respectively [10].

However, studies have not investigated whether using the SC type classification, based on body configuration, organ functions, or personality would increase the accuracy of risk prediction for metabolic syndrome. Therefore, using a 14-year longitudinal prospective cohort data from a general population of 3529 individuals (1769 men and 1760 women), we examined whether the SC types increase predictive accuracy.

## Methods

### Study population

An ongoing prospective cohort study, the Korean Genome Epidemiology Study (KoGES), was initiated in 2001. Detailed information on the study design and procedure has been previously reported [11]. At enrollment, the initial cohort of 10,030 participants, 40 to 69 years of age, was randomly recruited from two study sites, with 5012 participants from the urban community of Ansan (2518 men and 2494 women) and 5018 participants from the rural community of Ansung (2240 men and 2778 women). The participants were randomly selected from the general population by telephone, mail, and door-to-door visits. These participants have been biennially followed up. The participants underwent comprehensive tests, when visiting a research site, including physical examinations, biochemical and clinical examinations, and interviewer-administrated questionnaires. All participants provided written informed consent, and the study protocol was approved by the Human Subjects Review Committee at Korea University Ansan Hospital and Ajou University School of Medicine.

The SC type for each participant was identified from 2009 to 2012. Of 6878 eligible participants, 5840 were successfully classified by the SC type. Considering the SC type as a unique individual trait, we estimated the risk of developing a newly diagnosed metabolic syndrome according to the SC types for approximately 14 years from 2001 to 2014. At baseline, of 5840 participants who were classified into an SC type, we excluded 1860 adults who already had metabolic syndrome. Fifty-nine individuals were further excluded when diagnosed with an established cardiovascular disease. Additionally, fifty-six individuals were excluded due to missing data from any of covariates at the baseline examination (age; *n* = 1, sex; *n* = 1, body mass index (BMI); *n* = 1, education; *n* = 27, and glucose *n* = 26). Additionally, 336 participants were excluded for missing one or more follow-up examinations over the 14 years of the study. Therefore, 3529 participants (1769 men and 1760 women) were involved in the final analyses.

### Classification of Sasang constitutional types

A new diagnostic model was used to identify the subjects’ SC types. Detailed information on this new diagnostic model has been previously reported [12]. Briefly, this is based on a probability model using multivariable logistic regression with individual data on facial image, body shape, voice features, and questionnaire results. Specifically, facial images obtained with a digital camera were processed to extract the variables of facial characteristics. Eight circumference measurements, at the forehead, neck, axilla, chest, ribs, waist, pelvis, and hips, were measured to process the variables of body shape. The variables of voice features were processed with the Hidden Markov Model Toolkit (Cambridge University Engineering Department, Cambridge, United Kingdom) and the Praat voice analysis program (University of Amsterdam, Amsterdam, Netherlands). The questionnaire consisted of 67 multiple-choice questions about general temperament, eating habits, and physiological symptoms, and was processed for the variables of personality characteristics and physiological symptoms. Since no participants were classified into the TY type, the SC types in this study involved just three types, TE, SE, and SY.

### Definition of metabolic syndrome

Metabolic syndrome was defined according to the criteria of the National Cholesterol Education Program Adult Treatment Panel III [13]. Participants were identified with newly diagnosed metabolic syndrome if they had at least three out of the five following components: abdominal obesity (waist circumference for Asia-Pacific adults ≥90 cm for men and ≥80 cm for women) [14], elevated triglycerides (triglyceride ≥150 mg/dl), low HDL cholesterol (<40 mg/dl for men and <50 mg/dl for women), high blood pressure (systolic/diastolic pressure ≥ 130/85 mmHg or treatment with anti-hypertensive drugs), and elevated fasting blood glucose (≥110 mg/dl or treatment with anti-diabetic drug).

### Other measurements

Following a standardized protocol, professionally trained interviewers and health professionals help all participants undergo anthropometric examinations. Height and weight of the participants were measured in light clothes with no shoes. Each participant’s BMI was calculated as weight divided by height (kg/m^2^). After resting five minutes in a seated position, participants had blood pressure measurements taken with a mercury sphygmomanometer (Baumanometer®, W.A. Baum Co., Inc., Copiague, NY, USA). After fasting at least eight hours overnight, participants underwent an early morning blood sample collection. The blood samples were delivered for assays at the Seoul Clinical Laboratory (Seoul, Korea). In this lab, the levels of triglycerides (TG), HDL cholesterol, and fasting glucose were assessed using a chemistry analyzer (ADVIA 1650, Siemens, Tarrytown, NY).

### Statistical analysis

Continuous and categorical variables were examined using generalized linear models and chi-square tests. Multiple comparisons were conducted with Scheffe’s post hoc tests. Cox proportional hazard regression models were established to estimate hazard ratios (HRs) and 95% confidence intervals (CIs) for the risk of developing metabolic syndrome associated with the SC types. To compare the risk prediction models for metabolic syndrome, four models were established. Model 1 was adjusted for age, sex, BMI, education, income, smoking, drinking, and physical activity. Model 2 was further adjusted for total cholesterol and C-reactive protein (C-RP). Model 3 was additionally adjusted for fasting glucose, HDL cholesterol, triglyceride levels, and systolic and diastolic blood pressures. Model 4 was further adjusted for SC type. Estimated risks for the development of metabolic syndrome according to the SC types were calculated and displayed. To identify significant predictors according to each SC type, the forward selection method was used. The areas under the curve (AUC) from the receiver operating characteristic (ROC) according to the SC types were estimated to compare the accuracy of the risk prediction for incident cases of metabolic syndrome. Statistical analysis was performed with SAS version 9.4 (SAS Institute, Cary, North Carolina, USA). All *p*-values <0.05 were considered statistically significant.

## Results

### General characteristics of the study participants

**Fig. 1:**
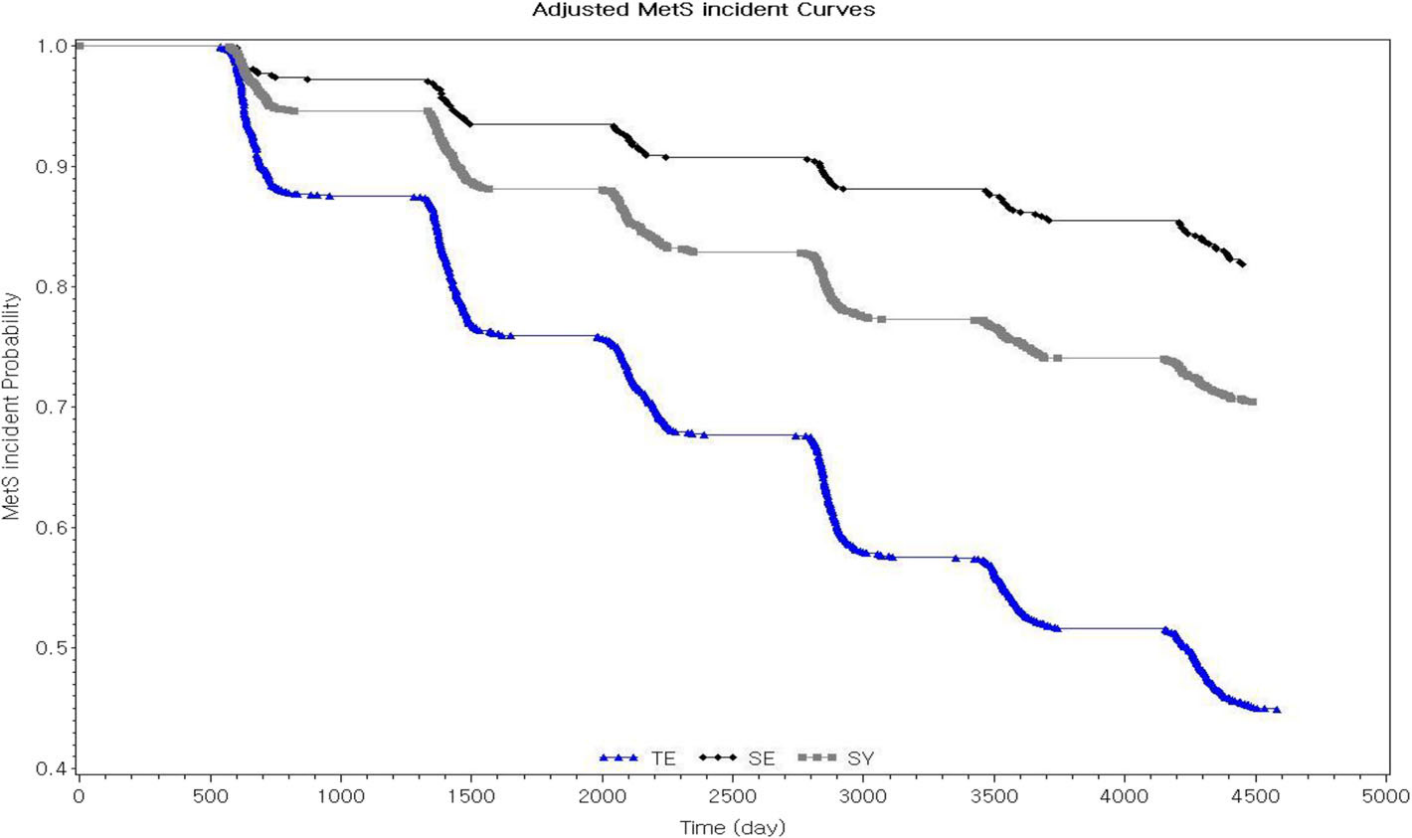
Estimated risk of the incident metabolic syndrome (*n* = 3529). The risk was adjusted for age, sex, BMI, education, income, smoking, drinking, and physical activity

Table 1 shows the general characteristics of the study participants. Of 3529 participants, the prevalence rates of each SC type were as follows: 45.03% (*n* = 1589) were TE, 13.43% (*n* = 474) were SE, and 41.54% (*n* = 1466) were SY. The TY type was not identified among the participants. The average ages, in years, of the study participants according to the SC types were 49.83 ± 8.08 for TE, 48.62 ± 7.57 for SE, and 49.76 ± 7.93 for SY. Women accounted for 46.51% of TE, 52.11% of SE, and 52.80% of SY individuals. The SC type had a significant association with education, income, smoking, and drinking. Specifically, individuals with the TE type showed a greater proportion of high education (>9 years of school), high income (>4 × 10^6^ won, annual income), current smokers (26.18%), and heavy drinkers (>30 g of ethanol consumption per day). However, participants, according to SC types, showed no difference in physical activity levels. For biochemical and clinical measurements, individuals with the TE type had higher levels of total cholesterol, C-RP, fasting glucose, systolic and diastolic blood pressures, and triglycerides than two other types. However, those with the TE type had a lower level of HDL cholesterol. Furthermore, the risks of incident metabolic syndrome according to SC type were evaluated after adjusting for age, sex, BMI, education, income, smoking, drinking, and physical activity (Fig. 1). The risk of developing metabolic syndrome was the highest among individuals with the TE type, followed by the SY type and the SE type.

**Table 1:** General Characteristics of the Study Participants (*n* = 3529)

	TE	SE	SY	*p*-value
	*n* = 1589 (45.03%)	*n* = 474 (13.43%)	*n* = 1466 (41.54%)	
Age, years	49.83 ± 8.08 ^a^	48.62 ± 7.57 ^b^	49.76 ± 7.93 ^a^	0.011
Women, n (%)	739 (46.51)	247 (52.11)	774 (52.80)	0.001
Body Mass Index, kg/m^2^	25.63 ± 2.47 ^a^	21.32 ± 1.98 ^b^	23.02 ± 2.17 ^c^	<0.001
Waist circumference, cm	83.85 ± 7.10 ^a^	73.87 ± 6.39 ^b^	77.52 ± 6.89 ^c^	<0.001
Education, years				
≤ 9	1288 (81.06)	390 (82.28)	1259 (85.88)	0.002
> 9	301 (18.94)	84 (17.72)	207 (14.12)	
Income, won				
< 2 × 10^6^	855 (53.81)	270 (56.96)	863 (58.87)	0.002
2 ~ 4 × 10^6^	552 (34.74)	173 (36.50)	475 (32.40)	
> 4 × 10^6^	182 (11.45)	31 (6.54)	128 (8.73)	
Smoking, n (%)				
Never smokers	888 (55.88)	298 (62.87)	914 (62.35)	0.001
Past smokers	285 (17.94)	63 (13.29)	231 (15.76)	
Current smokers	416 (26.18)	113 (23.84)	321 (21.90)	
Alcohol drinking, n (%)				
Non-drinkers	710 (44.68)	280 (59.07)	796 (54.30)	<0.001
Light (0.1 ~ 15)	511 (32.16)	128 (27.00)	408 (27.83)	
Moderate (15.1 ~ 30)	172 (10.82)	35 (7.38)	127 (8.66)	
Heavy (>30)	196 (12.33)	31 (6.54)	135 (9.21)	
Physical activity, MET-hours	19.97 ± 84.79 ^a^	14.79 ± 55.67 ^a^	16.54 ± 75.17 ^a^	0.310
Total-cholesterol, mg/dl	194.10 ± 35.07 ^a^	182.59 ± 32.94 ^b^	185.25 ± 32.63 ^b^	<0.001
C-reactive protein, mg/dl	0.22 ± 0.41 ^a^	0.18 ± 0.36 ^a^	0.19 ± 0.32 ^a^	0.032
Fasting Glucose, mg/dl	86.03 ± 16.33 ^a^	81.66 ± 9.16 ^b^	83.63 ± 14.07 ^c^	<0.001
HDL-cholesterol, mg/dl	45.92 ± 9.30 ^a^	47.76 ± 10.46 ^b^	47.20 ± 10.13 ^b^	<0.001
Systolic blood pressure, mmHg	116.74 ± 15.57 ^a^	113.04 ± 13.99 ^b^	114.42 ± 16.07 ^b^	<0.001
Diastolic blood pressure, mmHg	78.24 ± 10.41 ^a^	74.90 ± 8.99 ^b^	76.20 ± 10.30 ^c^	<0.001
Triglyceride, mg/dl	144.02 ± 87.86 ^a^	122.57 ± 64.98 ^b^	128.78 ± 70.38 ^b^	<0.001

mean ± SD; If the mean difference was significant, Scheffe post hoc test was utilized for multiple comparisons

Means with the same letter are indicated to be not significantly different

### Comparison of the risk prediction models for incident metabolic syndrome according to the Sasang constitutional types

**Fig. 2:**
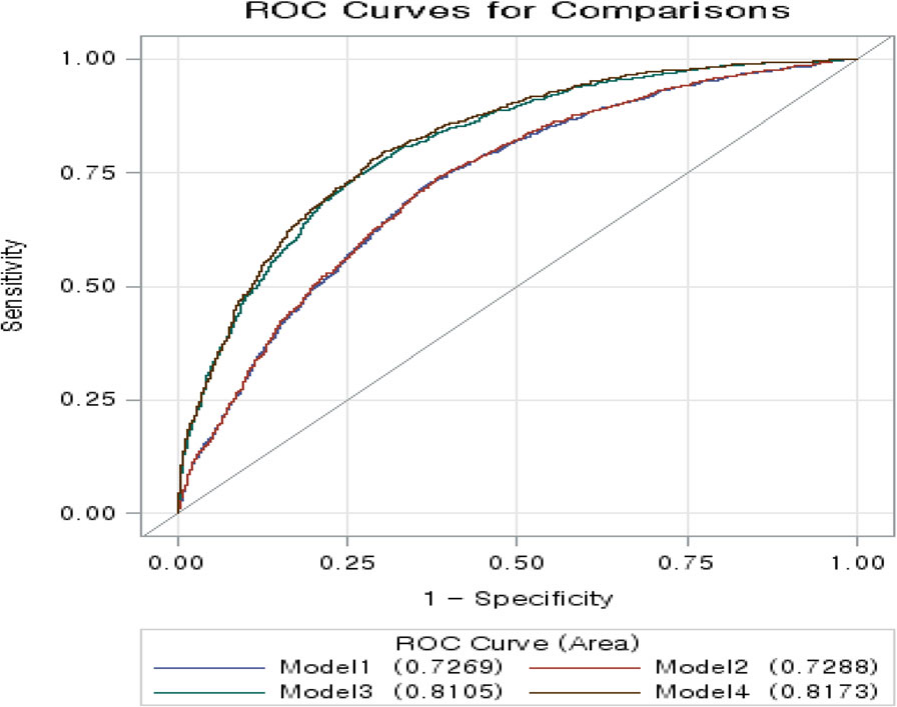
ROC curve (area) according to the models. Model 1 was adjusted for age, sex, body mass index, education, income, smoking, drinking, and physical activity; Model 2 was further adjusted for total cholesterol and C-reactive protein; Model 3 was additionally adjusted for fasting glucose, HDL cholesterol, triglyceride levels, and systolic and diastolic blood pressures; Model 4 was further adjusted for SC type

Table 2 presents four prediction models to compare the accuracy of the prediction risk for developing metabolic syndrome, and Fig. 2 shows the ROC curves. Model 1 had traditional risk factors for metabolic syndrome, and the AUC was 0.7269. Model 2 added two additional biochemical measurements, total cholesterol and C-RP levels, and the AUC was 0.7288. Model 3 additionally had metabolic syndrome components, such as fasting glucose, HDL cholesterol levels, systolic and diastolic blood pressures, and triglyceride levels, and the AUC was 0.8105. Finally, Model 4, utilizing the SC types, showed that the AUC was significantly increased to 0.8173.

**Table 2:** Risk Prediction Models of the Sasang Constitution (*n* = 3529)

	Model 1	Model 2	Model 3	Model 4
Sasang type				
TE				2.03 (1.63, 2.54)
SY				1.33 (1.07, 1.65)
SE				Reference
Age	1.04 (1.03, 1.04)	1.04 (1.03, 1.04)	1.03 (1.02, 1.04)	1.03 (1.02, 1.04)
Women	1.39 (1.18, 1.65)	1.39 (1.18, 1.64)	2.54 (2.13, 3.03)	2.55 (2.14, 3.04)
Body Mass Index	1.20 (1.18, 1.22)	1.19 (1.17, 1.21)	1.17 (1.15, 1.19)	1.11 (1.09, 1.14)
Education				
≤ 9 years	Reference	Reference	Reference	Reference
> 9 years	0.86 (0.74, 1.00)	0.84 (0.72, 0.98)	0.85 (0.73, 0.99)	0.83 (0.71, 0.97)
Income, won				
< 2 × 10^6^	1.07 (0.89, 1.29)	1.09 (0.91, 1.32)	1.08 (0.90, 1.31)	1.12 (0.93, 1.35)
2 ~ 4 × 10^6^	0.94 (0.78, 1.13)	0.95 (0.79, 1.15)	1.00 (0.83, 1.21)	1.03 (0.85, 1.24)
> 4 × 10^6^	Reference	Reference	Reference	Reference
Smoking				
Never smoker	Reference	Reference	Reference	Reference
Past smoker	1.09 (0.90, 1.32)	1.07 (0.88, 1.30)	1.01 (0.83, 1.23)	1.01 (0.83, 1.23)
Current smoker	1.52 (1.28, 1.80)	1.50 (1.26, 1.79)	1.70 (1.42, 2.03)	1.67 (1.40, 1.99)
Alcohol drinking				
Non-drinker	Reference	Reference	Reference	Reference
Light (0-15 g/d)	1.01 (0.90, 1.14)	1.01 (0.89, 1.13)	1.09 (0.97, 1.24)	1.06 (0.94, 1.20)
Moderate (15–30 g/d)	1.08 (0.89, 1.31)	1.08 (0.89, 1.31)	1.09 (0.90, 1.33)	1.04 (0.86, 1.27)
Heavy (>30 g/d)	1.30 (1.08, 1.56)	1.28 (1.07, 1.54)	1.36 (1.13, 1.65)	1.32 (1.09, 1.60)
Physical activity, MET-hours	1.00 (1.00, 1.00)	1.00 (1.00, 1.00)	1.00 (1.00, 1.00)	1.00 (1.00, 1.00)
Total-cholesterol, mg/dl		1.00 (1.00, 1.00)	1.00 (1.00, 1.01)	1.00 (1.00, 1.01)
C-reactive protein, mg/dl		1.07 (0.95, 1.21)	1.05 (0.92, 1.20)	1.05 (0.92, 1.20)
Fasting glucose, mg/dl			1.01 (1.01, 1.02)	1.01 (1.01, 1.02)
HDL-cholesterol, mg/dl			0.95 (0.95, 0.96)	0.95 (0.95, 0.96)
Systolic blood pressure, mmHg			1.02 (1.02, 1.03)	1.02 (1.02, 1.03)
Diastolic blood pressure, mmHg			1.01 (1.00, 1.02)	1.01 (1.00, 1.02)
Triglyceride, mg/dl			1.00 (1.00, 1.00)	1.00 (1.00, 1.00)
Area Under the Curve (AUC)	0.7269	0.7288	0.8105	0.8173
*p*-value for the area under the ROC curve	<0.001	<0.001	0.001	Reference

The risks for metabolic syndrome were estimated in HR (95% CIs) that was Hazard Ratio (HR) and 95% Confidence Intervals (CIs)

### Predictors for the development of metabolic syndrome according to the Sasang constitutional types

Table 3 indicates significant predictors for incident metabolic syndrome according to the SC types. Using a forward selection method to identify significant predictors of metabolic syndrome, individuals with the TE type showed significant predictors in age, sex, BMI, education, smoking, drinking, fasting glucose level, HDL cholesterol level, systolic and diastolic blood pressure, and triglyceride levels. Individuals with the SE type demonstrated significant predictors in sex, smoking, fasting glucose, HDL cholesterol, systolic and diastolic blood pressure, and triglyceride. Individuals with the SY type indicated significant predictors in age, sex, BMI, smoking, drinking, total cholesterol, fasting glucose, HDL cholesterol, systolic and diastolic blood pressure, and triglycerides.

**Table 3:** Selected Predictors according to Sasang Types

	TE	SE	SY
Age	1.03 (1.02, 1.04)		1.04 (1.03, 1.05)
Women	2.13 (1.70, 2.67)	4.65 (2.17, 9.99)	3.42 (2.51, 4.65)
Body Mass Index	1.12 (1.09, 1.15)		1.12 (1.08, 1.17)
Education			
≤ 9 years	Reference		
> 9 years	0.78 (0.65, 0.94)		
Income, won			
< 2 × 10^6^			
2 ~ 4 × 10^6^			
> 4 × 10^6^			
Smoking			
Never smoker	Reference	Reference	Reference
Past smoker	1.05 (0.82, 1.34)	0.69 (0.23, 2.11)	1.05 (0.74, 1.48)
Current smoker	1.62 (1.29, 2.03)	1.90 (0.88, 4.08)	1.78 (1.31, 2.42)
Alcohol drinking			
Non-drinker	Reference		Reference
Light (0-15 g/day)	1.17 (1.01, 1.36)		0.97 (0.78, 1.22)
Moderate (15–30 g/day)	0.88 (0.68, 1.13)		1.43 (1.02, 2.01)
Heavy (>30 g/day)	1.35 (1.07, 1.69)		1.25 (0.88, 1.80)
Physical activity, MET-hours			
Total-cholesterol, mg/dl			1.01 (1.00, 1.01)
C-reactive protein, mg/dl			
Fasting glucose, mg/dl	1.01 (1.01, 1.01)	1.03 (1.02, 1.05)	1.01 (1.01, 1.02)
HDL-cholesterol, mg/dl	0.96 (0.95, 0.97)	0.96 (0.93, 0.98)	0.95 (0.94, 0.96)
Systolic blood pressure, mmHg	1.03 (1.02, 1.03)	1.04 (1.02, 1.06)	1.02 (1.01, 1.03)
Diastolic blood pressure, mmHg	1.01 (0.99, 1.02)	1.01 (0.98, 1.04)	1.02 (1.01, 1.04)
Triglyceride, mg/dl	1.00 (1.00, 1.00)	1.01 (1.00, 1.01)	1.00 (1.00, 1.00)

Significant predictors were selected with a forward selection method. The risks for metabolic syndrome were estimated in HR (95% CIs) that was Hazard Ratio (HR) and 95% Confidence Intervals (CIs)

## Discussion

In a 14-year longitudinal study among 3529 individuals, we found that utilizing the SC types of the participants significantly increased the accuracy of the risk prediction for incident metabolic syndrome. Particularly, an increase in the accuracy of the risk prediction using the SC types was observed in a model that already had traditional risk factors and sub-components of metabolic syndrome.

It is important to predict the risk of developing metabolic syndrome in order to prevent cardiometabolic disorders, because metabolic syndrome has been known to increase the risk of type 2 diabetes and cardiovascular diseases [8]. A previous study demonstrated that the SC type was a significant risk factor for metabolic syndrome [15, 16]. Additionally, individuals of the TE type have an increased risk for metabolic syndrome as the number of components of metabolic syndrome increased [16]. Consistent with previous studies [10, 15, 16], our finding demonstrated that the TE type was the most prevalent, accounting for 45.03% of participants, and was associated with a greater risk of developing metabolic syndrome than the other types (OR = 2.03, 95% CI 1.63, 2.54). Individuals with the TE type, who have been known to have a greater predisposition to metabolic syndrome, are characterized with larger waist circumferences and higher frequencies of smoking and heavy drinking [1], as confirmed in our findings. In contrast, individuals with the SE type indicated a reduced risk of developing metabolic syndrome than the other types [10].

With more than 100 years of accumulated experience and empirical methods, the SC types of a traditional Korean medicine, initiated by Jema Lee (1837–1900), specifies individual’s physical features including facial color and shape, voice, personality, behavioral tendency, and other characteristics [1]. Thus, the SC types have allowed a distinct advantage in identifying disease susceptibilities. For example, individuals with the same disease would have a different prognosis and treatment, depending on each patient’s SC type.

The underlying mechanism on how the SC types increase the accuracy of the risk prediction for incident metabolic syndrome in a general population has not been clearly explained. However, recent evidence has been accumulated on susceptible organs and vulnerable diseases according to the SC types including the recent systematic review [6]. Future studies are warranted to examine the phenotypic characteristics of each SC type.

This study has strengths and a limitation. First, the study participants were randomly selected from a general population through telephone contacts, mails, and door-to-door visits. The general population has no particular characteristics, so that the results might be applicable to others. Additionally, this study was based on a 14-year longitudinal cohort study that allows us to assess causal relationship. However, this study has a limitation to consider when interpreting our results. A sub-study of the SC types was initiated from the fifth wave (year 2009/2010). Thus, the study participants of the SC type were only based on the number of the participants who visited at the fifth and six waves.

## Conclusions

In conclusion, in a 14-year longitudinal prospective cohort study among 3529 individuals (1769 men and 1760 women), we observed that utilizing the SC types significantly contributed to increasing the accuracy of predicting the risk of developing metabolic syndrome, though the increment was small. Based on our observation that SC type played a significant role in predicting the risk, the SC types may need to be included into a risk prediction model for metabolic syndrome.

## Abbreviations

CI: Confidence interval
HDL: High-density lipoprotein
HR: Hazard ratio
ROC: Receiver operating characteristic
SC: Sasang constitution
SCM: Sasang constitutional medicine
SE: So-eum
SY: So-yang
TE: Tae-eum
TY: Tae-yang
